# Brief psychosocial education, not core stabilization, reduced incidence of low back pain: results from the Prevention of Low Back Pain in the Military (POLM) cluster randomized trial

**DOI:** 10.1186/1741-7015-9-128

**Published:** 2011-11-29

**Authors:** Steven Z George, John D Childs, Deydre S Teyhen, Samuel S Wu, Alison C Wright, Jessica L Dugan, Michael E Robinson

**Affiliations:** 1 Department of Physical Therapy, PO Box 100154, University of Florida, Gainesville, FL, 32610 USA; 2 Department of Physical Therapy (MSGS/SGCUY), 81st Medical Group, Keesler Air Force Base, Biloxi, MS, 39534 USA; 3 US Army-Baylor University Doctoral Program in Physical Therapy (MCCS-HMT), Army Medical Department Center and School, 3151 Scott Rd., Rm. 2307, Fort Sam Houston, TX 78234 USA; 4 US Army Public Health Command Region-South, Fort Sam Houston, TX 78234 USA; 5 Department of Biostatistics, PO Box 117450, University of Florida, Gainesville, FL 32611 USA; 6 Department of Clinical and Health Psychology, PO Box 100165, Health, University of Florida, Gainesville, FL 32610 USA; 7 Center for Pain Research and Behavioral Health, PO Box 100165, University of Florida, Gainesville, FL, 32610 USA

**Keywords:** primary prevention, core stabilization, patient education, incidence, low back pain

## Abstract

**Background:**

Effective strategies for the primary prevention of low back pain (LBP) remain elusive with few large-scale clinical trials investigating exercise and education approaches. The purpose of this trial was to determine whether core stabilization alone or in combination with psychosocial education prevented incidence of low back pain in comparison to traditional lumbar exercise.

**Methods:**

The Prevention of Low Back Pain in the Military study was a cluster randomized clinical study with four intervention arms and a two-year follow-up. Participants were recruited from a military training setting from 2007 to 2008. Soldiers in 20 consecutive companies were considered for eligibility (n = 7,616). Of those, 1,741 were ineligible and 1,550 were eligible but refused participation. For the 4,325 Soldiers enrolled with no previous history of LBP average age was 22.0 years (SD = 4.2) and there were 3,082 males (71.3%). Companies were randomly assigned to receive traditional lumbar exercise, traditional lumbar exercise with psychosocial education, core stabilization exercise, or core stabilization with psychosocial education, The psychosocial education session occurred during one session and the exercise programs were done daily for 5 minutes over 12 weeks. The primary outcome for this trial was incidence of low back pain resulting in the seeking of health care.

**Results:**

There were no adverse events reported. Evaluable patient analysis (4,147/4,325 provided data) indicated no differences in low back incidence resulting in the seeking of health care between those receiving the traditional exercise and core stabilization exercise programs. However, brief psychosocial education prevented low back pain episodes regardless of the assigned exercise approach, resulting in a 3.3% (95% CI: 1.1 to 5.5%) decrease over two years (numbers needed to treat (NNT) = 30.3, 95% CI = 18.2 to 90.9).

**Conclusions:**

Core stabilization has been advocated as preventative, but offered no such benefit when compared to traditional lumbar exercise in this trial. Instead, a brief psychosocial education program that reduced fear and threat of low back pain decreased incidence of low back pain resulting in the seeking of health care. Since this trial was conducted in a military setting, future studies are necessary to determine if these findings can be translated into civilian populations.

**Trial Registration:**

NCT00373009 at ClinicalTrials.gov - http://clinicaltrials.gov/

## Background

Musculoskeletal pain, and especially low back pain (LBP), adversely affects military preparedness as common reasons for medical evacuation [1] with return to duty being uncertain [1,2]. Furthermore, LBP is also a common reason for long-term Soldier disability [3]. It is not surprising then that prevention of LBP remains a high research priority for the general [4] and military societies [1,2].

Effective strategies for preventing LBP remain elusive. Physical exercise has consistent evidence for primary prevention of LBP compared to no activity [5], but a review for the European Guidelines for Prevention of Low Back Pain indicated there were not enough studies to allow for recommendations differentiating types of exercise [6]. Back schools, lumbar supports and ergonomic interventions have limited support in systematic reviews [5,7], and, therefore, are not recommended for primary prevention of LBP [6]. Education for primary prevention of LBP has received mixed support in trials [5]; there has been some support for psychosocial education, but not for biomedical or biomechanical based education programs [6]. Priorities for LBP prevention research noted in the European Guidelines included higher quality randomized trials that investigated specific physical exercise interventions in combination with psychosocial education [6].

The Prevention of Low Back Pain in the Military (POLM) cluster randomized clinical trial incorporated core stabilization exercise because of its preventative potential [8,9]. We also incorporated psychosocial education based on the Fear-Avoidance Model of Musculoskeletal Pain (FAM) [10,11]. Earlier POLM studies reported our core stabilization program was associated with shorter work restriction from LBP [12], and the psychosocial education program resulted in a positive shift in Soldier back beliefs [13]. Planned future analyses of the POLM trial include investigation of how core stabilization exercise affects activation of key lumbar musculature, predictors of first episode of LBP, and an economic analysis of these interventions.

The current paper then reports on the primary findings of the POLM cluster randomized trial. The POLM trial had four intervention arms consisting of traditional lumbar exercise, traditional lumbar exercise with psychosocial education, core stabilization exercise, and core stabilization exercise with psychosocial education groups. These intervention groups were compared for their effects in preventing LBP during two years of military duty. The POLM trial's aims were consistent with previously mentioned primary prevention priorities and we investigated individual level effects of exercise and education programs. We hypothesized that Soldiers receiving core stabilization and psychosocial education would have lower incidence of LBP in comparison to those receiving only traditional lumbar exercise.

## Methods

The institutional review boards at the Brooke Army Medical Center (Fort Sam Houston, Texas) and the University of Florida (Gainesville, FL) granted ethical approval for this project. All Soldiers provided written informed consent prior to their participation. A more detailed description of the POLM trial protocol has been previously published [14]. Data in this paper were reported in compliance with the Consolidated Standards of Reporting Trials (CONSORT) guidelines extension for cluster randomized trials [15].

### Subjects

Consecutive Soldiers entering a 16-week training program at Fort Sam Houston, TX to become combat medics in the U.S. Army were considered for participation in the POLM trial from February 2007 to March 2008. This training program occurred after completion of basic training.

Subjects were required to be 18 to 35 years of age (or 17-year-old emancipated minors) and be able to speak and read English. Subjects with a prior history of LBP were excluded. A prior history of LBP was operationally defined as LBP that limited work or physical activity, lasted longer than 48 hours, and caused the subject to seek health care. Subjects were also excluded if they were currently seeking medical care for LBP; unable to participate in unit exercise due to musculoskeletal injury; had a history of lower extremity fracture (stress or traumatic); were pregnant; or had transferred from another training group. Other possible exclusions included Soldiers who were being accelerated into a company already randomized or Soldiers who were being re-assigned to a different occupational specialty.

### Exercise programs

Subjects performed the assigned group exercise program under the direct supervision of their drill instructors as part of daily unit physical training. Specifically, the entire company exercised at the same time with each individual platoon being led by one of six drill sergeants assigned to a particular platoon for the training period. Therefore, these exercise programs are likely to pertain to individual, platoon and company levels. The traditional exercise program (TEP) was selected from commonly performed exercises for the rectus abdominus and oblique abdominal muscles. These exercises are routinely performed inside (and outside) the military environment and are utilized to assess physical performance of Soldiers [16]. Core stabilization exercise approaches differ in that they target deeper trunk muscles that attach to the spine; such as the transversus abdominus, multifidus and the erector spinae. These muscles play a key supportive role that contribute to the ability of the lumbar spine to withstand loading [17,18] and exercises that target these muscles are believed to have preventative effects for LBP [8,9]. The core stabilization exercise program (CSEP) used in the POLM trial consisted of exercises shown with potential to selectively activate these same muscle groups to directly test these purported preventative effects. The TEP and CSEP are described in Table 1 and in more detail in previous POLM publications [12,16]. The TEP was an active comparison treatment condition because a no-exercise intervention group was not feasible in the military environment.

**Table 1:** Description of core stabilization (CSEP) traditional (TEP) and exercise programs

Exercise	CSEP	TEP
**Principle**	Lower load, less repetitions	Higher load, more repetitions
**Activation**	Slower	Faster
**Trunk movements**	None to minimal	Full
**Dosage**	Five minutes/day	Five minutes/day
#1	Abdominal drawing-in maneuver crunch	Traditional sit-up
#2	Left and right horizontal side support	Sit-up with left trunk rotation
#3	Hip flexor squat	Sit-up with right trunk rotation
#4	Supine shoulder bridge	Abdominal crunch
#5	Quadruped alternate arm and leg	Traditional sit-up

CSEP, core stabilization exercise program; TEP, traditional exercise program

The TEP and CSEP exercise regimens consisted of five to six exercises, each of which was performed for one minute. Exercise programs were performed daily, for a total dosage time of five minutes per day, five days per week over 12 weeks. Study personnel monitored physical training an average of two days per week over the 12-week training period to answer questions and monitor compliance with the assigned exercise program.

### Brief education program

The brief psychosocial education program (PSEP) involved attendance at one session during the first week of training. For the education program, the company was divided into two or three groups to accommodate the size of the lecture hall and also to allow for flexibility in scheduling Soldiers. Each group received the same information and the session involved an interactive lecture led by study personnel (ACW, JLD) lasting approximately 45 minutes. The lecture consisted of a visual presentation followed by a question and answer session. The PSEP provided Soldiers current, evidence-based information on LBP that was designed to reduce its threat and fear, such as stressing that anatomical causes of LBP are not likely to be definitely identified and encouraging active coping strategies. Educational material was provided by issuing each Soldier *The Back Book *for personal use as has been done in previous trials [19-21]. The PSEP is described in more detail in a previous POLM publication [13]. We did not include a control education program as prior studies consistently demonstrated comparison education approaches did not favorably alter LBP beliefs [19,20].

### Randomization

Military training environments require living in close quarters with other members of the unit, making individual randomization an unfeasible option due to treatment contamination. Therefore, a cluster randomization strategy was utilized as this is a viable methodological choice for large primary prevention trials [22,23]. The POLM trial had four intervention arms comprised of a combination of the previously described exercise and education programs. The specific intervention combinations for cluster random assignment included TEP only, TEP + PSEP, CSEP only, and CSEP + PSEP.

The randomization schedule was prepared by computer and determined before recruitment began. The randomization schedule was balanced to ensure that equal number of companies was allocated to each program. Treatment allocation was done in a concealed manner at the University of Florida and this process was supervised by our lead statistician (SSW). The randomly generated intervention groups were completed prior to study recruitment and listed in sequential order. This list was then stored on a secure server at the University of Florida. When a new cohort of Soldiers was scheduled to start their 12-week training program the study coordinators at Brooke Army Medical Center (ACW, JLD) contacted research personnel at the University of Florida for the appropriate intervention assignment.

### Blinding

It was not possible to mask Soldiers because they actively participated in the exercise and education training programs. All outcomes were assessed by raters blinded to group assignment or were obtained via self-report.

### Baseline measures

Measures were collected under supervision of research personnel unaware of random company assignment and scored in a masked manner by computer algorithm. Soldiers completed standard demographic information, such as age, sex, past medical history, and factors related to military status. Soldiers also completed self-report measures at baseline for physical and mental function [24], anxiety [25], depressive symptoms [26], fear of pain [27], and back beliefs [28].

### Outcome measures

We originally intended to assess self-report of LBP incidence using a web-based data collection system, in which Soldiers were reminded by email to complete on-line forms about whether they had experienced LBP in the last calendar month [14]. However, one year follow-up rates were much lower than anticipated (18.4%) [29]. Exact reasons for the low follow-up rate from the self-report method were unknown but it could have been due to deployment to Iraq or Afghanistan limiting ability to access the web-based system. At one year follow-up a decision was made to instead measure LBP incidence by tracking Soldiers that sought healthcare for LBP. Therefore, the primary outcome for this study is best conceptualized as incidence of LBP that resulted in the seeking of healthcare. This decision to change the method of measuring incidence was based solely on concerns with low follow-up rates noticed before the primary study endpoint [29]. The study team made the decision without the benefit of preliminary analyses and health care utilization was not originally a secondary outcome. Furthermore, only a health care utilization database was considered as the means to generate an alternate measure for LBP incidence. The decision to use a health care utilization database to measure LBP incidence was reinforced when the final two-year self-report response rate remained low at 1,230/4,325 (28.4%).

The Military Health System (MHS) Management Analysis and Reporting Tool (M2 database) was used to determine LBP incidence mainly because of its comprehensive nature in capturing health care utilization. Our interest in using a health care seeking definition of experiencing LBP was driven by studies indicating continuing high rates of health care utilization for LBP [30,31] with trends of greatly increasing cost, but of no obvious benefit to the population [32,33]. In addition, the validity of self-report measures for determining LBP has been questioned for military populations [34], and use of a health care database mitigated these concerns. The M2 database is maintained by the Tricare Management Activity of the MHS and contains a variety of health care data regarding patient care from both the direct care system (care provided in military treatment facilities) and network care (care provided to MHS beneficiaries at civilian facilities) worldwide. Additionally, the data collected to populate the M2 database includes healthcare use while Soldiers are deployed to such areas as Iraq or Afghanistan. The M2 database was searched for relevant LBP-related International Classification of Diseases (ICD) codes for Soldiers enrolled in the POLM trial. We used similar strategies to operationally define LBP as has been published in other studies, using ICD codes to identify subjects seeking health care for LBP [35,36]. We had originally planned to investigate the severity of the first LBP episode but the M2 database did not include measures that allowed for such an estimate. Therefore, the severity of LBP outcome measure was abandoned from the reporting of POLM trial primary results.

### Sample size estimation and power analysis

This trial intended to recruit a minimum of 16 companies based on the assumption of 150 consenting Soldiers per company. A more detailed sample size estimation and power analysis was published with our trial protocol [14].

### Data analysis

There were no planned interim analyses or stopping rules for the POLM trial [14]. All statistical analyses were performed using the SAS software, version 9 (SAS Institute Inc, Cary, North Carolina, United States,1996). Demographic and baseline levels of clinical variables were compared among the four intervention groups using analysis of variance (ANOVA) for means and chi-square tests for proportions. Variables that differed between the four intervention groups were considered in the final analyses, in addition to pre-specified covariates of gender and age.

The incidence of LBP resulting in the seeking of health care data was analyzed with a generalized linear mixed model and the response variable was the number of months in which a Soldier reported LBP. Because this was a cluster randomized trial we considered company as a random effect. The planned fixed effects were treatment group, age and gender, as well as any variables that differed among the four intervention groups after randomization. Survival time to the first day of LBP was investigated with a Cox proportional hazards model and log-rank test to investigate treatment effects. The response variable was time to first day in which treatment for LBP was identified in the M2 database using the date of enrollment as the starting point. The predictor variables for the survival analysis were the same variables included in the generalized linear mixed models.

## Results

**Figure 1:**
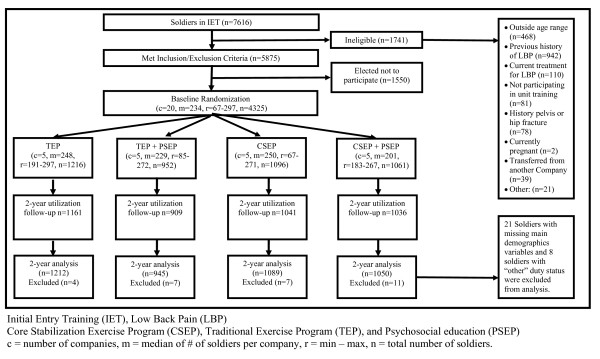
**Flow diagram for patient recruitment and randomization**.

Figure 1 provides information on study enrollment, assignment to the four intervention arms, participation, follow-up, and analysis for all stages of the POLM trial [15]. There were no reported adverse events for the education and exercise programs. Table 2 provides baseline characteristics for each of the randomly assigned exercise and education combinations. Baseline differences across individuals in the four companies were found in age, education, income, active duty status and time in the army (Table 2). These differences were controlled for in subsequent analyses and, therefore, all data from the regression models are presented as adjusted estimates.

**Table 2:** Comparison of baseline characteristics across the intervention groups

**Variable**	**Label**	**Overall**	**TEP**	**TEP+PSEP**	**CSEP**	**CSEP+PSEP**	***P*-Value**
			
		N = 4,325	N = 1,216	N = 952	N = 1,096	N = 1,061	
Innate characteristics							
**A Age**		22.0 ± 4.2	21.6 ± 4.1	22.6 ± 4.5	21.8 ± 4.0	22.1 ± 4.3	<0.0001
**Gender**	Male	3,082	870 (71.7%)	689 (72.5%)	758 (69.5%)	765 (72.7%)	0.335
	Female	1,226	344 (28.3%)	262 (27.5%)	333 (30.5%)	287 (27.3%)	
**Race**	Black or Africa	420	104 (8.6%)	88 (9.3%)	114 (10.4%)	114 (10.8%)	0.236
	Hispanic	426	128 (10.5%)	97 (10.3%)	115 (10.5%)	86 (8.1%)	
	White or Caucas	3,190	897 (73.8%)	711 (75.2%)	797 (72.8%)	785 (74.1%)	
	Other	279	86 (7.1%)	50 (5.3%)	69 (6.3%)	74 (7.0%)	
**Education**	High school or lower	1,935	600 (49.3%)	409 (43.0%)	484 (44.2%)	442 (41.7%)	0.0038
	Some college	1,998	504 (41.4%)	463 (48.6%)	506 (46.2%)	525 (49.5%)	
	College or higher	391	112 (9.2%)	80 (8.4%)	105 (9.6%)	94 (8.9%)	
**Income**	Less than $20,000	2,125	620 (51.2%)	418 (44.0%)	583 (53.3%)	504 (47.7%)	0.0001
	Greater than $20,000	2,188	592 (48.8%)	532 (56.0%)	511 (46.7%)	553 (52.3%)	
**Active Duty**	Active	2,532	725 (59.6%)	504 (52.9%)	737 (67.4%)	566 (53.4%)	<0.0001
	Reserve	1,782	491 (40.4%)	446 (46.8%)	356 (32.5%)	489 (46.1%)	
	Other	8		2 (0.2%)	1 (0.1%)	5 (0.5%)	
**Time In Army**	<5 months	2,691	768 (63.2%)	566 (59.5%)	737 (67.4%)	620 (58.5%)	<0.0001
	5 months to 1 year	969	276 (22.7%)	198 (20.8%)	222 (20.3%)	273 (25.8%)	
	More than 1 year	661	172 (14.1%)	188 (19.7%)	134 (12.3%)	167 (15.8%)	
**Height**		68.3 ± 3.9	68.4 ± 3.7	68.4 ± 3.8	68.1 ± 4.0	68.4 ± 4.0	0.340
**Weight**		164.8 ± 27.7	164.8 ± 26.7	165.7 ± 28.2	163.8 ± 27.9	165.2 ± 28.0	0.426
**BMI**		24.8 ± 3.1	24.7 ± 3.0	24.8 ± 3.3	24.7 ± 3.2	24.7 ± 3.2	0.807
Psychological							
**BDI Total**		6.4 ± 6.6	6.5 ± 6.9	6.4 ± 6.7	6.5 ± 6.5	6.3 ± 6.2	0.843
**FPQ Total**		18.1 ± 5.9	17.8 ± 5.9	18.2 ± 5.9	18.0 ± 6.1	18.2 ± 5.6	0.317
**BBQ Total**		43.4 ± 7.1	43.3 ± 7.2	43.1 ± 6.9	44.0 ± 6.8	43.2 ± 7.2	0.010
**STAI**		36.0 ± 9.1	36.2 ± 9.5	35.8 ± 9.1	35.7 ± 9.0	36.3 ± 9.0	0.337
Baseline health status and physical activity							
**SF 12 PCS**		53.4 ± 5.2	53.7 ± 5.0	53.5 ± 5.0	53.4 ± 5.3	53.1 ± 5.2	0.041
**SF 12 MCS**		49.2 ± 8.6	49.2 ± 8.7	49.1 ± 8.7	49.2 ± 8.5	49.0 ± 8.5	0.938
**Smoke Prior to Army**	Yes	1,552	442 (36.3%)	354 (37.2%)	374 (34.2%)	382 (36.0%)	0.534
	No	2,771	774 (63.7%)	598 (62.8%)	720 (65.8%)	679 (64.0%)	
**Exercise Routinely**	Yes	2,220	627 (51.6%)	474 (49.8%)	560 (51.2%)	559 (52.7%)	0.647
	No	2,102	589 (48.4%)	477 (50.2%)	534 (48.8%)	502 (47.3%)	
Attention/Relational Effect							
**Physical Exam**	No	3,951	1,128 (92.8%)	855 (89.8%)	1,005 (91.7%)	963 (90.8%)	0.087
	Yes	374	88 (7.2%)	97 (10.2%)	91 (8.3%)	98 (9.2%)	

BBQ, Back Beliefs Questionnaire; BDI, Beck Depression Inventory; BMI, body mass index; CSEP, core stabilization exercise program; FPQ, Fear of Pain Questionnaire (9 items); PSEP, psychosocial education program; SF 12 MCS, Mental Component Summary Score from the Short Form Medical Survey (12 items); SF 12 PCS, Physical Component Summary Score from the Short Form Medical Survey (12 items); STAI, State Trait Anxiety Inventory (state portion only); TEP, traditional exercise program

### Low back pain incidence resulting in seeking of health care

Over two years the number of Soldiers captured in the M2 database was 4,147/4,325 (95.9%), and, of those, 706 (17.0%) had LBP resulting in seeking of health care. Lower incidence of LBP resulted from the combination of any exercise with education (CSEP + PSEP and TEP + PSEP). Table 3 shows LBP incidence by percentage for all 20 individual companies (coefficient of intracluster correlation of 0.0053). Table 3 also shows the incidence data by the four randomly assigned intervention groups on which the primary analyses were completed.

**Table 3:** LBP rate by company based on utilization data

Training Group	Company	N	Number (%) of Soldiers with LBP incidence resulting in seeking of health care
**TEP**	1	191	30 (15.7%)
	2	252	41 (16.3%)
	3	228	37 (16.2%)
	4	297	59 (19.9%)
	5	248	46 (18.5%)
	**All**	**1216**	**213 (17.5%)**
**TEP + PSEP**	1	272	36 (13.2%)
	2	85	12 (14.1%)
	3	229	39 (17.0%)
	4	103	15 (14.6%)
	5	263	30 (11.4%)
	**All**	**952**	**132 (13.9%)**
**CSEP**	1	250	44 (17.6%)
	2	271	33 (12.2%)
	3	239	50 (20.9%)
	4	269	55 (20.4%)
	5	67	11 (16.4%)
	**All**	**1096**	**193 (17.6%)**
**CSEP + PSEP**	1	217	37 (17.1%)
	2	183	26 (14.2%)
	3	193	29 (15.0%)
	4	201	35 (17.4%)
	5	267	41 (15.4%)
	**All**	**1061**	**168 (15.8%)**
**CSEP**	Yes	2157	361(16.7%)
	No	2168	345 (15.9%)
**PSEP**	Yes	2013	300 (14.9%)
	No	2312	406 (17.6%)

Data presented in the table are unadjusted. Total intervention groups are in bold. The intracluster correlation coefficient (ICC) is 0.0053. For the test of equal LBP rate across intervention groups, comparing TEP Yes vs No, and comparing PSEP Yes vs No, the Chi-Square values equal to 6.99, 0.54, and 5.56; with corresponding *P*-values of 0.0722, 0.4641 and 0.0183, respectively. Adjusting for ICC, the Chi-square values reduce to 6.35, 0.49, and 5.05; with corresponding *P*-values of 0.0957, 0.482 and 0.0246, respectively

The analyses of the four intervention groups suggested a pattern that allowed for more efficient communication of results by collapsing the intervention groups into those receiving any core stabilization (CSEP - yes or no) or any psychosocial education (PSEP - yes or no). There were no differences between the TEP + PSEP and CSEP + PSEP groups, but chi-square test indicated that receiving the PSEP program with any exercise program was protective of LBP incidence (Chi-square = 5.56, *P *= 0.018; and 5.05, *P *= 0.025 when adjusted for intracluster correlation) in comparison to those not receiving PSEP. Furthermore, after adjusting for demographic and baseline levels of clinical variables, the protective pooled effect of any PSEP was estimated at 3.3% (95% CI: 1.1 to 5.5%) decreased LBP incidence over two years (*P *= 0.007). This effect corresponds to numbers needed to treat (NNT) of 30 (95% CI = 18.2 to 90.9).

**Figure 2:**
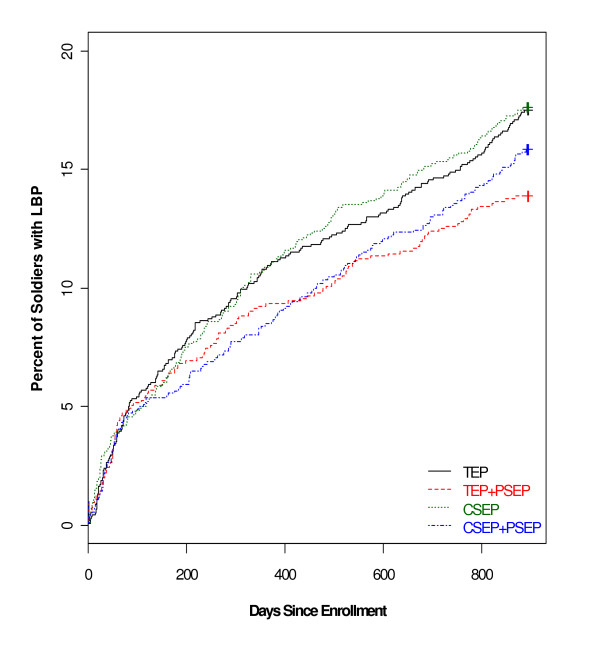
**Percent of Soldiers reporting incidence of low back pain (unadjusted data)**.

Results from the generalized linear mixed model indicated that Soldiers in the combined exercise and psychosocial education groups (CSEP + PSEP and TEP + PSEP) were similar, but experienced an average of 0.49 fewer months with incidence of LBP (95% CI: 0.003 to 0.983, *P *= 0.048) in comparison to those not receiving PSEP. Survival analysis on the time to the first day of LBP demonstrated a similar pattern (Figure 2), where the preventative effect of any psychosocial education was observed (hazard ratio = 0.90; Log-Rank test, *P *= 0.021).

## Discussion

The POLM cluster randomized trial is the first large scale trial to test the purported primary prevention effects of core stabilization, alone and in combination with psychosocial education, for LBP. Trial results suggest no benefit of core stabilization exercises for preventing LBP incidence resulting in the seeking of health care in comparison to traditional lumbar exercises. In contrast, a brief psychosocial education program in combination with either of the exercise programs resulted in lower two-year incidence of health care-seeking for LBP. These results have potential importance for primary prevention strategies for Soldiers in the military given the high rates of evacuation due to musculoskeletal pain and injuries that adversely affects Soldier preparation [1,2].

The overall decrease in LBP from brief psychosocial education might be perceived as small, but the 3.3% decrease represented the absolute risk reduction, whereas the relative risk reduction was approximately 17%. Furthermore, seeking health care for LBP is very common [30,31], so even small decreases in LBP incidence could potentially lessen the burden on a health care system. The psychosocial education program was administered in a single, low-cost session. There is potential for similar education programs to be done in an efficient manner, such that when applied to populations they yield incremental decreases in LBP incidence. Prevention of health care seeking by education seems especially relevant when increased usage and expenditures of health care for LBP have not resulted in obvious improvements in population outcomes [32,33].

The primary limitation of the current study is that these results may have limited direct application to civilian populations due to trial implementation in a military setting. For example, an alternate explanation for the null effects of core stabilization exercise could be that Soldiers in this trial were at high levels of general fitness and not likely to benefit from additional exercise. Another limitation is that the current study did not include a true control condition so we cannot comment on the absolute effects of the exercise programs. We did have a randomly selected group of Soldiers who received additional attention from a physical examination and ultrasound imaging [14]. There were no differences in LBP incidence for these Soldiers, suggesting no general attention effect in this trial (Table 2).

The decision to shift from a self-report definition of LBP incidence to a definition based on seeking of health care is another limitation to consider. As previously noted, this decision was made before the planned end of the study, was not based on any interim analyses, and was not a process of choosing one outcome from multiple potential outcomes. However, the end result of this decision is that our incidence measure of LBP resulting in the seeking of health care was not based on self-report of symptoms and had close to 96% follow-up at two years. There is the potential that these findings could underestimate the effect of these interventions on mild LBP episodes that did not necessitate health care and also we were not able to further describe the utilization of health care. For example, we could not distinguish between services that were provided for care during the episode. Overall, however, we feel the shift to a LBP incidence definition that accounted for health care seeking provided an unintended positive dimension to the POLM trial. The individual differences after cluster randomization could have led to systematic effects based on the company, rather than the assigned education program. However, we had low intracluster correlations suggesting independence between clusters and outcome measure. Baseline cluster differences were also small in magnitude (Table 2) and we accounted for company as a random effect in all analyses. Therefore, we are confident that individual cluster effects are fully accounted for when presenting the results.

Another weakness of this study is that Soldiers did additional sit-ups to prepare for fitness testing and this training could have adversely affected the core stabilization exercise [12,16]. However, the rate of additional sit-ups was equivalent across the four groups so any additive effects of extra training would likely have had an equal impact on outcomes. We took a pragmatic approach to exercise dosing and it could be argued that dosage parameters for core stabilization were not sufficient to generate a preventative effect. However, our dosing parameters were consistent with expert recommendations for core stabilization exercise [37]. Furthermore, we did not facilitate or track exercise performance of any kind after the 12-week training period and that is another weakness to consider. Finally, we did not determine if the LBP episode resulted in medical board (disability) or evacuation for Soldiers with LBP and this outcome measure would be of importance for future prevention studies.

A strength of the POLM trial is that we recruited a large inception cohort of Soldiers not previously experiencing LBP. This factor was highlighted as a research priority for LBP prevention studies in the European Guidelines [6] and the application of potentially preventative interventions before deployment was consistent with recent military recommendations [1,2]. Two-year follow-up of all LBP episodes is an additional strength of the POLM trial. Finally, use of a health care utilization database to define LBP incidence is a strength of the study because of increased utilization trends for LBP [30-33] and concerns with using self-report definitions in military samples [34]. Readers should realize, however, that this was a specific way of determining LBP incidence and the results of the POLM trial may not generalize to other ways of determining LBP incidence (for example, survey methods).

Exercise and education for primary prevention of LBP has received mixed support from the European Guidelines [6] and systematic reviews of work place interventions [5,38]. Individual trials have suggested some types of exercise may be preventative of LBP when compared to no intervention [39], but similar effects have been reported when exercise was compared to patient education [40]. In the POLM trial, two different exercise approaches targeting trunk musculature were compared and there was no benefit from performing specific core stabilization as we had hypothesized. The POLM trial findings are, therefore, consistent with Guideline recommendations [6] that indicate no added benefit of a particular focused exercise approach for prevention of LBP. Future studies investigating primary prevention of LBP may consider different methods for delivering exercise, such as tailored individualized approaches that have demonstrated efficacy for treatment of patients with chronic LBP [41].

The POLM trial did provide data indicating that psychosocial education based on the FAM has potential value for decreasing incidence of LBP resulting in the seeking of health care. Similar positive effects for LBP of psychosocial patient education based on the FAM have been reported in quasi-experimental studies in Australia [42] and France [21]. Although there is some evidence that FAM factors have limited prognostic value in acute stages of LBP [43], these educational studies provide evidence of benefit either before pain [42] or in the acute stage of LBP [21]. What the previously reported education studies do not often address is processes that may account for the benefit. In the case of the POLM trial, we did perform a planned preliminary analysis to investigate the short term efficacy of our psychosocial education program for a proximal endpoint that occurred after their 12-week training but before deployment [13]. In this preliminary analysis, Soldiers receiving the psychosocial education program reported improved beliefs related to the inevitable consequences of LBP as measured by the Back Beliefs Questionnaire [13]. In contrast, Soldiers not receiving the psychosocial education program had a slight worsening of their beliefs of LBP. It, therefore, could be asserted that a positive shift in beliefs about LBP while an individual is pain-free may result in decreased likelihood to seek health care when LBP was later experienced during military deployment. This earlier study provides data to support a process to explain the primary findings of the POLM trial, but we did not collect LBP beliefs with the Back Beliefs Questionnaire during the episode of LBP, so we lack the long term data that would directly validate this process.

There are unanswered questions and future research directions to consider following the POLM trial. Future studies could consider testing the preventative capability of core stabilization in different populations with lower overall fitness levels. Also, determining if the psychosocial education program translates to different civilian settings would be of particular interest as there are other trials that have demonstrated positive shifts in LBP beliefs for school age children [44] and older nursing home residents [45]. This particular psychosocial education program used in the POLM trial has potential to generate cost-savings for those seeking health care for LBP, especially if it prevents exposure to expensive interventions that have questionable efficacy [32]. Finally, we used what could be considered a small dose of psychosocial education with no reinforcement after the initial session [13]. Different dosages and reinforcement strategies for the education program could be explored in future studies to determine if larger effect sizes are observed for primary prevention of LBP.

## Conclusions

The European Guidelines for Prevention of Low Back Pain [6] indicated a high priority for rigorous randomized clinical trials that investigate primary prevention of LBP. Completion of the POLM trial meets this priority and has provided additional data for those interested in primary prevention of LBP. Specifically, our results suggest that exercise programs that target core lumbar musculature may offer no additional preventative benefit when compared to traditional lumbar exercise programs. Also, brief psychosocial education may be an important adjunct to exercise programs as they may prevent the seeking of health care when experiencing LBP. These are novel findings and, since this study was done in a military setting, future research is necessary to determine whether these education programs could be implemented in civilian populations with similar efficacy. In addition, future studies should consider the cost-benefit of education programs that reduce LBP incidence resulting in the seeking of health care.

## Abbreviations

CONSORT: Consolidated Standards of Reporting Trials; CSEP: core stabilization exercise program; FAM: Fear-Avoidance Model of Musculoskeletal Pain; ICD: International Classification of Diseases; LBP: low back pain; MHS: Military Health System; NNT: numbers needed to treat; POLM: Prevention of Low Back Pain in the Military; PSEP: psychosocial education program; TEP: traditional exercise program

## Competing interests

The authors have no competing interests to declare with submission of this manuscript. All authors have completed the Unified Competing Interest form at http://www.icmje.org/coi_disclosure.pdf and these forms are available on request from the corresponding author. All authors received financial support from the Department of Defense to complete the submitted work; have no financial relationships with any organizations that might have an interest in the submitted work in the previous 3 years; and have no other relationships or activities that could appear to have influenced the submitted work.

## Authors' contributions

SZG, JDC, DST, SSW and MER were responsible for the initial conception of the research question, securing funding, supervising the protocol, and final manuscript preparation. SSW was primarily responsible for data analysis, interpretation and reporting, while SZG, JDC, DST and MER assisted with interpretation and reporting. ACW and JLD were responsible for implementing the study protocol. All authors read, edited and approved the final version of the manuscript.

## Pre-publication history

The pre-publication history for this paper can be accessed here:

http://www.biomedcentral.com/1741-7015/9/128/prepub
